# Early Motor Developmental Milestones and Schizotypy in the Northern Finland Birth Cohort Study 1966

**DOI:** 10.1093/schbul/sbx165

**Published:** 2017-12-09

**Authors:** Svetlana Filatova, Heli Koivumaa-Honkanen, Golam M Khandaker, Estelle Lowry, Tanja Nordström, Tuula Hurtig, Kristiina Moilanen, Jouko Miettunen

**Affiliations:** 1 Center for Life Course Health Research, University of Oulu, Finland; 2 Medical Research Center Oulu, Oulu University Hospital and University of Oulu, Finland; 3 Institute of Clinical Medicine (Psychiatry), University of Eastern Finland, Finland; 4 Department of Psychiatry, Kuopio University Hospital, Finland; 5 South-Savonia Hospital District, Finland; 6 North Karelia Central Hospital, Finland; 7 SOTE, Finland; 8 Lapland Central Hospital, Finland; 9 Department of Psychiatry, University of Cambridge, UK; 10 Cambridgeshire and Peterborough NHS Foundation Trust, UK; 11 Biocenter Oulu, University of Oulu, Finland; 12 Center for Clinical Neurosciences, Department of Psychiatry, University of Oulu, Finland; 13 PEDEGO Research Unit, Child Psychiatry, University of Oulu, Finland; 14 Clinic of Child Psychiatry, University Hospital of Oulu, Finland; 15 Department of Psychiatry, Oulu University Hospital, Finland

**Keywords:** motor milestone, schizotypy, birth cohort

## Abstract

Delayed motor developmental milestones have been reported to be associated with schizophrenia in previous studies, but no study has examined the relationship between early motor developmental milestones and schizotypy. We have examined this relationship in a prospective birth cohort.In the Northern Finland Birth Cohort 1966, data on 9 early motor developmental milestones were collected prospectively from visits to child welfare centers, and data on adult schizotypy were collected through a questionnaire (*N* = 4557–4674). Positive schizotypy was measured by the Perceptual Aberration Scale (PAS), negative schizotypy was measured by Physical Anhedonia Scale (PhAS) and Social Anhedonia Scale (SAS). Three related scales were included: Schizoidia Scale (SCHD), Hypomanic Personality Scale (HPS), and Bipolar II Scale (BIP2). We examined the milestone–schizotypy associations before and after excluding cases of schizophrenia from this population-based sample. Hierarchical regression analyses adjusted for covariates and separately for both genders were performed. In men, each extra month of delay in achievement of touching thumb with index finger, sitting unsupported, standing up, walking with support, or walking unsupported was associated with an increase in PAS, PhAS, or SCHD scores, or decrease in BIP2 score (*P* < .05). In women, each extra month of delay in achievement of turning from back to tummy was associated with an increase in PhAS and SAS scores (*P* < .05). Schizotypy is associated with delayed motor developmental milestones in early-life, but there is some heterogeneity with regards to types of milestones and gender. These findings suggest delayed motor development confers risk across the continuum of schizophrenia syndrome.

## Introduction

The link between schizotypy and motor abnormalities has been suggested based on several findings. Among that evidence is higher prevalence of neurological soft signs in individuals with either psychometrically identified schizotypy^1^ or proneness to schizotypal personality disorder (SPD).^2^ Furthermore, excessive motor force and its higher variability have been found during experimental tasks in adults with SPD.^3^ Also poor motor performance on the line drawing task has been linked with psychometrically measured schizotypy.^4^ While there is some evidence on the association between early-life events (ie, placental weight, birth weight, head circumference, etc.) and schizotypy,^5^ research regarding the early risk factors of schizotypy have remained scarce.

Schizotypy can be defined a complex of personality traits including peculiar behavior, odd beliefs, and reduced positive affect^6,7^ as well as a dynamic continuum that range from personality to psychosis.^8^ Schizotypy can be assessed by positive (ie, cognitive and perceptual disturbances) and negative (asociality, anergia) dimensions,^9,10^ which parallel schizophrenia symptoms. Individuals with pronounced schizotypy have an increased risk of developing schizophrenia.^6,7,9,11^ In a 10-year longitudinal general population study, Kwapil et al^8^ found that individuals with high positive and high negative schizotypy had 1.50 (95% CI = 1.16–1.94) and 1.87 (95% CI = 1.37–2.55) times higher risk of any-schizophrenia spectrum disorder, respectively. Furthermore, genetic findings indicate, for instance, that schizotypy traits measured by Perceptual Aberration Scale (PAS), Social Anhedonia Scale (SAS), Physical Anhedonia Scale (PhAS), and Schizoidia Scale (SCHD) were associated with alleles in DISC1 gene related to schizophrenia.^12^ This and other early-life period findings suggest the link between schizophrenia and schizotypy.

Since schizophrenia has been suggested to be a neurodevelopmental disorder,^5,13,14^ this theory can also be applied for schizotypy etiology. It is known that schizophrenia and schizotypy share a number of early life risk factors, eg, maternal infection, maternal diabetes, maternal smoking during pregnancy, obstetric complications, low birth weight, neurological soft signs, low socioeconomic status (SES), and winter/autumn birth.^8,15,16^ Motor abnormalities are another common early risk factor between schizophrenia and schizotypy,^1,4,17–22^ and have been suggested to be early sign of schizophrenia liability.^23–25^ Also early motor developmental milestones, ie, walking, sitting, or standing unsupported, have been linked in a meta-analysis with subsequent schizophrenia,^26^ but have not yet been studied in respect to schizotypy. In this, the risk factors relevant to positive or negative dimensions of schizotypy should be clearly differentiated, but this has not been done previously.

Previously, in NFBC 1966, an association between schizophrenia and later achievement of standing without support or walking without support was found only in men.^27^ Furthermore, higher vulnerability of a brain and predisposition to neurodevelopmental anomalies was found in men who developed schizophrenia compared to women.^27–30^ Lahti et al^5^ showed gender difference also in types of early risk factor-schizotypy associations. Lastly, according to a meta-analysis,^31^there was gender difference also in the prevalence of schizotypal traits. Men had higher negative schizotypy (ie, physical and social anhedonia), but no gender differences were observed in positive schizotypy (ie, perceptual aberration). These findings support that associations between milestones and schizotypy should be studied separately in both genders.

Research is needed to identify whether early motor developmental milestones play a similar role in etiology of schizotypy as in schizophrenia. The aim of the present study was to investigate in the large NFBC 1966 the association between early motor developmental milestones and schizotypy taking into account gender and schizotypy dimensions. We hypothesized that delay in early motor developmental milestones would be associated with increase in both positive and negative dimensions of schizotypy.

## Methods

### Sample

Data were derived from the 31-year follow-up of the NFBC 1966 (*n* = 12 058 live births) followed prospectively since mother’s pregnancy. A cohort subject was excluded if she/he (1) had mental disability (ICD-8: 310–315, ICD-9: 317–319, ICD-10: F70–F79), (2) scored more than 3 on the Infrequency scale which identifies random response patterns, (3) denied access to data, (4) was a twin, and (5) had all milestones and all schizotypy scales data missing (supplementary figure 1). The data for psychoses were collected from several nationwide registers: (1) Care Register for Health Care; (2) Finnish outpatient registers; (3) Social Insurance Institution registers: reimbursable medicines, sick days, and disability pensions; (4) Finnish Centre for Pensions: disability pensions and diagnosed according to ICD-8: 295, 2954, 2957, 2960–2969, 297, 298, 2980, 299; ICD-9: 295, 2954, 2957, 2961E, 2962E, 2963E, 2964E, 2967, 297, 2988, 2989; or ICD-10: F20, F22-24, F25, F28-29, F302, F312, F315, F323, F333 and previously reported.^26,32^ The most recent data on psychoses and mental disability were obtained from the 47 year follow-up (December 2013) by linkage with all available health registers. Schizotypy can be both studied with individuals who developed clinical psychosis and without them. Thus, we compared the found results of associations between early motor developmental milestones and schizotypy if schizophrenia cases were included or not. The sample that included schizophrenia cases consisted of 4674 participants: 2602 women and 2072 men (supplementary table 1). There were 117 cases of psychosis, who fulfilled inclusion criteria and did not have missing data on at least one milestone and at least one scale (for full breakdown of diagnosis please see supplementary table 2). The sample with excluded schizophrenia cases consisted of 4557 participants: 2529 women and 2028 men. The number of missing data varied depending on scale or milestone being studied (supplementary figure 1). Thus, the sample sizes varied in different analyses, which are also reported in table 1.

**Table 1.** Multivariate Linear Regression Analyses of 9 Early Motor Developmental Milestones and Schizotypal Scales by Gender

	Schizophrenia Cases Included						Schizophrenia Cases Excluded					
	Women			Men			Women			Men		
Scale/Milestone	*B* ^a^	95 % CI	*P* Value	*B*	95 % CI	*P* Value	*B*	95 % CI	*P* Value	*B*	95 % CI	*P* Value
PAS^b^	*N* = 917–2393			*N* = 722–1908			*N* = 903–2333			*N* = 709–1868		
Walking with support	0.02	−0.08 to 0.11	0.754	**0.12**	**0.03 to 0.22**	**0.012**	0.02	−0.08 to 0.12	0.730	**0.09**	**0.00 to 0.19**	**0.044**
Capable to stand up (lift themselves)	0.06	−0.07 to 0.19	0.331	**0.14**	**0.01 to 0.28**	**0.038**	0.07	−0.06 to 0.20	0.277	0.07	−0.06 to 0.20	0.278
Touching thumb with index finger (like a tweezer)	0.11	−0.05 to 0.27	0.167	**0.25**	**0.09 to 0.42**	**0.002**	0.11	−0.05 to 0.27	0.178	**0.22**	**0.06 to 0.37**	**0.006**
PhAS	*N* = 1444–1582			*N* = 1139–1337			*N* = 1421–1559			*N* = 1114–1307		
Capable to stand up (lift themselves)	0.09	−0.13 to 0.31	0.446	**0.36**	**0.04 to 0.68**	**0.026**	0.08	−0.14 to 0.30	0.479	0.31	−0.01 to 0.63	0.057
Sitting unsupported	0.17	−0.11 to 0.43	0.224	**0.39**	**0.02 to 0.78**	**0.040**	0.17	−0.10 to 0.44	0.221	0.35	−0.03 to 0.73	0.068
Turning from back to tummy	**0.32**	**0.06 to 0.58**	**0.017**	0.05	−0.32 to 0.42	0.789	**0.35**	**0.08 to 0.61**	**0.010**	0.03	−0.34 to 0.41	0.858
SAS	*N* = 1440–1577			*N* = 1137–1335			*N* = 1417–1554			*N* = 1114–1307		
Sitting unsupported	**0.27**	**0.05 to 0.49**	**0.015**	−0.11	−0.41 to 0.19	0.464	**0.26**	**0.05 to 0.48**	**0.049**	−0.16	−0.46 to 0.14	0.305
Turning from back to tummy	**0.31**	**0.09 to 0.53**	**0.005**	−0.05	−0.35 to 0.25	0.743	**0.30**	**0.08 to 0.52**	**0.007**	−0.07	−0.37 to 0.23	0.646
SCHD	*N* = 921–1583			*N* = 724–1336			*N* = 907–1560			*N* = 711–1308		
Touching thumb with index finger (like a tweezer)	−0.01	−0.08 to 0.05	0.682	**0.09**	**0.02 to 0.16**	**0.017**	−0.01	−0.08 to 0.05	0.681	**0.08**	**0.01 to 0.16**	**0.008**
Turning from back to tummy	0.02	−0.04 to 0.09	0.480	**0.09**	**0.02 to 0.16**	**0.011**	0.02	−0.04 to 0.08	0.54	0.07	−0.00 to 0.14	0.057
BIP2	*N* = 1423–1642			*N* = 1183–2042			*N* = 1400–1993			*N* = 1160–1610		
Walking unsupported	−0.06	−0.17 to 0.05	0.273	−0.13	−0.25 to −0.00	0.047	−0.06	−0.17 to 0.05	0.253	**−0.15**	**−0.28 to −0.02**	**0.021**
Making a grip on object (grab object)	−0.14	−0.42 to 0.14	0.325	**−0.33**	**−0.64 to −0.02**	**0.038**	−0.16	−0.44 to 0.12	0.256	−0.30	0.02 to −0.05	0.063

*Note:* Adjusted for parental psychoses, parental age, place of residence and father’s socioeconomic status. 95% CI, 95% confidence interval; PAS, Perceptual Aberration Scale; PhAS, Physical Anhedonia Scale; SAS, Social Anhedonia Scale; SCHD, Schizoidia Scale; BIP2, Bipolar 2 Scale.

The bold values indicate *P* < 0.05.

^a^ *B* refers to unstandardized regression coefficient derived from linear regression analyses.

^b^Adjusted also for birth weight.

### Early Motor Developmental Milestones

Data on 9 early motor developmental milestones were collected from visits to Finnish child welfare centers. These milestones included walking unsupported, standing unsupported, walking with support, capable to stand up (lift themselves), touching thumb with index finger, sitting unsupported, turning from back to tummy, make a grip on an object (grab object), holding the head up. The achievement of milestones was assessed by medical personnel at approximately monthly intervals^32^ and recorded on a separate welfare card as a standard practice in Finland. The data on milestones were collected at an age of at least 11.5 month or later in 96% of cases.^33^ The mean number of appointments during the first year was 10.^27^ If a participant missed an appointment, then information on milestones were recorded during the next visit retrospectively.

### Schizotypy

Among scales measuring schizotypal traits, positive schizotypy is measured by the Perceptual Aberration Scale (PAS) and negative schizotypy by the Social Anhedonia Scale (SAS) and the Physical Anhedonia Scale (PhAS).^6,34,35^ Furthermore, SAS, PAS, and PhAS have shown a strong predictive ability for psychosis.^9,36,37^ This is also true for the Hypomanic Personality Scale (HPS) and the Schizoidia Scale (SCHD).^37^ Lastly, an overlap between schizoaffective and bipolar disorder was found,^38^ and therefore the Bipolar II Scale (BIP2)^39^ was also included.

Thus, the 6 scales in the present study were 3 scales measuring schizotypy: (1) PAS with 35 items,^35^ (2) revised PhAS^34^ with 61 items, and (3) SAS^34^ with 40 items; and 3 related scales: (4) HPS^40^ with 48 items, (5) SCHD with 7 items,^41^ and (6) BIP2^39^with 31 items.

High scores on schizotypy scales characterize a personality with distorted perception of one own’s body and other objects (PAS), lack of interest in social interaction (SAS), and a lowered ability to experience physical and sensory pleasures (PhAS).^34,35^ High scores on schizotypy-related scales describe tireless and irritable individuals (HPS), prone to bipolar disorder (BIP2) and having psychotic traits (SCHD).^39–41^ The questionnaire, consisting of scales with true/false questions (scored 0/1), was administered to individuals in the 31-year follow-up. The scales have been translated from English to Finnish and backtranslated.

### Covariates

We selected the following available potential confounders based on prior research indicating that they are likely to be associated with schizophrenia and schizophrenia-related outcomes: parental psychosis, father’s SES, place of residence, and parental age.^42,43^ We have also considered birth weight, when studying motor milestones associations with PAS.^5^ Information regarding parental psychoses were obtained from nationwide registers and there were 272 (5.8%) individuals with a history of parental psychoses (supplementary table 1). The distribution of diagnoses was as follows: 84 schizophrenia narrow, 52 delusional disorder, 8 schizoaffective disorder, 22 bipolar disorder with psychotic features, 57 major depressive episodes with psychotic features, 18 brief psychoses, 24 other nonorganic psychoses, and 7 unspecified psychoses.

### Statistical Analysis

We studied schizotypy scales as continuous variables and the scales were examined for normality.^44^ Only PAS and SAS required transformation. Thus, PAS was log-transformed and SAS was square-root transformed.^44^ We report results both for transformed and original scales. Univariate linear and hierarchical linear regression analyses with covariates were done separately for both genders. In addition, all these analyses were performed with or without schizophrenia cases. To explore associations between covariates, exposures, and outcomes Pearson’s or polychoric correlations were applied.

To explore the effect of parental psychosis on association between early motor developmental milestones and schizotypy we conducted multivariate linear regression without individuals with history of parental psychosis. All analyses were performed in SPSS 21, except polychoric correlations, which were performed in R 3.3.2.

## Results

### Association Between Early Motor Developmental Milestones and Schizotypy When Schizophrenia Cases Are Included

In men, each extra month of delay in achievement of walking with support, capability to stand up, touching thumb with index finger were associated with a 0.12 (*R*^2^ = .010; *P* = .012), 0.14 (*R*^2^ = .007, *P* = .038) and 0.25 (*R*^2^ = .030, *P* = .002) increase and in PAS score (table 1). However, after the scale transformation only associations between walking with support and touching thumb with index finger remained significant (*R*^2^ = .007, *P* = .034; *R*^2^ = .015, *P* = .005; table 2). Each extra month of delay in achievement of the capability to stand up was associated with a 0.36 increase and sitting unsupported with a 0.39 increase in PhAS score (*R*^2^ = .010, *P* = .026, *R*^2^ = .012, *P* = .040). Also in men, each extra month of delay in achievement of touching thumb with index finger and turning from back to tummy were associated with a 0.09 increase in SCHD score (*R*^2^ = .019, *P* = .017; *R*^2^ = .024, *P* = .011). In contrast, a delay in achievement of walking unsupported and make a grip on object were associated with a 0.13 and 0.33 decrease in BIP2 score (*R*^2^ = .007, *P* = .047; *R*^2^ = .013, *P* = .038; table 1).

**Table 2.** Multivariate Linear Regression Analyses of Early Motor Developmental Milestones and PAS (Log-Transformed) and SAS (Square-Root Transformed) by Gender

	Schizophrenia cases included						Schizophrenia cases excluded					
	Women			Men			Women			Men		
Scale/Milestone	*B*	95% CI	*P* value	*B*	95% CI	*P* value	*B*	95% CI	*P* value	*B*	95% CI	*P* value
PAS^a^	*N* = 917–2393			*N* = 722–1908			*N* = 903–2333			*N* = 709–1868		
Walking with support	0.01	−0.01 to 0.14	0.329	0.01	0.00 to 0.21	0.034	0.01	−0.01 to 0.02	0.293	0.01	−0.00 to 0.02	0.070
Capable to stand up (lift themselves)	0.01	−0.00 to 0.02	0.108	0.13	−0.01 to 0.03	0.066	0.01	−0.00 to 0.24	0.088	0.01	−0.01 to 0.22	0.247
Touching thumb with index finger (like a tweezer)	0.01	−0.01 to 0.02	0.397	**0.03**	**0.01 to 0.04**	**0.005**	0.01	−0.01 to 0.02	0.474	**0.02**	**0.01 to 0.04**	**0.009**
SAS	*N* = 1440–1577			*N* = 1137–1335			*N* = 1417–1554			*N* = 1114–1307		
Sitting unsupported	0.04	−0.00 to 0.07	0.056	−0.02	−0.07 to 0.03	0.378	0.04	−0.00 to 0.07	0.061	−0.03	−0.07 to 0.02	0.244
Turning from back to tummy	**0.06**	**0.02 to 0.09**	**0.004**	−0.01	−0.05 to 0.04	0.701	**0.05**	**0.02 to 0.09**	**0.006**	−0.01	−0.06 to 0.03	0.581

*Note:* Adjusted for parental psychoses, parental age, place of residence, and father’s socioeconomic status.

The bold values indicate *P* < 0.05.

^a^Adjusted also for birth weight.

In women, each extra month of delay in achievement of turning from back to tummy was associated both with a 0.32 increase in PhAS (*R*^2^ = .013, *P* = .017) and a 0.31 increase in SAS (*R*^2^ = .017, *P* = .005). Each extra month delay in achievement of sitting unsupported was associated with 0.27 increase in SAS score (*R*^2^ = .016, *P* = .015), but only in the analysis with a nontransformed scale (table 2).

### Associations Between Early Motor Developmental Milestones and Schizotypy When Schizophrenia Cases Were Excluded

In men, each extra month of delay in achievement of walking with support and touching thumb with index finger were associated with a 0.09 (*R*^2^ = .011; *P* = .044) and 0.22 (*R*^2^ = .024, *P* = .006) increase and in PAS score (table 1). Only association between touching thumb with index finger and PAS remained significant in transformed scale analysis (table 2).

Each extra month delay in touching thumb with index finger was associated with 0.08 increase SCHD score (*R*^2^ = .018, *P* = .008) and each month delay in walking unsupported was associated with 0.15 decrease in BIP2 (*R*^2^ = .008, *P* = .021; table 1). Delay in milestones was not significantly associated with PhAS or SAS (table 1 and table 2).

In women, each extra month of delay in achievement of turning from back to tummy was associated both with a 0.35 increase in PhAS (*R*^2^ = .015, *P* = .010) and 0.30 increase in SAS (*R*^2^ = .017, *P* = .007). Each extra month delay in sitting unsupported was associated with 0.26 increase in SAS scale (*R*^2^ = .019, *P* = .049), but only in the analysis with a nontransformed scale (table 1 and table 2).

### Early Motor Developmental Milestones and Clinically Diagnosed Schizotypy When Schizophrenia Cases Included

There were 32 cases of clinically diagnosed schizotypy out of which 24 developed schizophrenia.

Men with clinically diagnosed schizotypy achieved walking with support, standing unsupported, and walking unsupported significantly later compared to those without diagnosis (*P* < .05) No differences were found in women.

### Associations Between Covariates, Exposures, and Outcomes (Schizophrenia Cases Included)

In men, advanced paternal age was associated with increase in PAS, SCHD, BIP2 scales and delay in all milestones, except sitting unsupported, turning from back to tummy, and holding the head up. Advanced maternal age was associated with increase in SCHD scale scores and with delay in all milestones, except holding the head up (results not shown).

In women, advanced paternal age was associated with delay in all milestones and increase in PAS, PhAS, SAS, and SCHD scale. Advanced maternal age was associated with increase in SAS scale and delay in all milestones, except making a grip on object and holding the head up (results not shown).

The results of correlations between categorical covariates (paternal psychosis, place of residence, and father’s SES) and milestones or scales showed that majority of significant correlations with exposures and outcomes were between place of residence or father’s SES in men and if schizophrenia cases were included (supplementary table 3).

### Influence of Parental Psychosis on Associations Between Early Motor Developmental Milestones and Schizotypy

We compared the results of significant milestones–scales associations with a study sample in which individuals with history of parental psychoses were either included or excluded. In the sample where individual schizophrenia cases were included, 2 associations became insignificant (supplementary table 4). When both individuals with schizophrenia and history of parental psychoses were excluded, 2 additional significant associations were found and 1 became insignificant (supplementary table 4).

## Discussion

Several milestones were associated with schizotypy at the age of 31 years, but this varied in regards to gender and the used scale. In general, a delay of milestone was associated with higher scores on scales related to positive (PAS) and negative schizotypy (SAS and PhAS), and on the SCHD scale, but in the BIP2 the association was negative.

### Schizotypy in Relation to Schizophrenia

Early motor developmental milestones (standing up, standing without support, walking without support) have also been previously linked with adult schizophrenia.^26^ In addition, Lahti et al^5^ found that several early life factors, eg, lower placental and birth weight, smaller head circumference at 12 months, and maternal smoking, were predictive for positive or negative schizotypy in men or women.

Thus, based on these previous NFBC 1966 studies, it was not surprising that in our study a delay in early motor developmental milestones was associated with increase in schizotypy. More significant milestones–schizotypy associations were found if individuals with schizophrenia were included (9 vs 5). It was logical that the sample that includes these severe cases was associated with more motor delays. However, the remained associations were between the same milestone-scale as in the analysis with schizophrenia cases. All this could further suggest a link between schizotypy and schizophrenia, even if some of their associated milestones might vary. Two milestones were associated with a decrease in BIP2 scores in men, which could suggest that earlier motor development might also sometimes even have negative impact on personality, or be at least associated for instance with lack of inhibition.^45^ However, as BIP2 significantly positively correlated with other schizotypal traits in both sexes (results not shown), this finding is probably by chance. Still, further research is needed.

Interestingly, some studies have suggested that abnormalities in neurodevelopmental markers are specific for negative dimensions of schizotypy.^1,46^ However, findings of the present study as well as a recent review by Barrantes-Vidal et al^11^ have showed that neurodevelopment deviations are also associated with positive dimensions of schizotypy. The milestones associated with clinically diagnosed schizotypy were the same that were associated previously with schizophrenia in meta-analysis.^26^ However, the sample size was small and diagnosis of schizotypy has not been validated. Thus, this research question requires improvement of the quality of the data and replication.

### Gender Differences in Associations Between Early Motor Developmental Milestones and Schizotypy

Higher scores on negative schizotypy, but lower scores on positive schizotypy were found in men compared to women. The results on gender differences in negative schizotypy are in line with a recent meta-analysis, but no differences on a positive schizotypy have been previously found.^31^

It has been reported that men at high risk of schizophrenia had poorer premorbid social functioning and greater externalizing behavior compared to women.^47,48^ Preschizophrenic women, but not men, followed since infancy to adolescence have expressed joy to a lower extent than their same sex siblings.^49^ According to another study, women suffering from schizophrenia had more adverse affective symptoms (ie, irritability, dysphoria, hostility, impulsivity, etc.), auditory hallucinations and delusions of being persecuted, while men had more negative symptoms (ie, poor speech, amotivation, social withdrawal, and blunted affect).^47^ In addition, the incidence of schizophrenia has been higher among men, but no gender differences have been found in the incidence of all psychoses.^48^ Therefore, it was suggested that men and women are predisposed to different subtypes of schizophrenia, the schizoaffective type being more common among women.^28^ Further, it was suggested that men are more vulnerable to neuromotor abnormalities than women.^27–30^ This can possibly explain why we found more early motor developmental milestone–schizotypy associations (ie, with both positive and negative schizotypy and psychotic traits) in men, while in women only two motor milestone–negative schizotypy association were found.

Interestingly, different types of milestones predicted different schizotypy traits in men and women. For example, walking with support was associated with PAS in men and turning from back to tummy with SAS and PhAS in women. However, the reasons behind gender differences in the types of milestones associated with schizotypy still need further research.

### Strengths and Limitations

To our knowledge, this is the first study exploring an association between early motor developmental milestones and schizotypy. It adds evidence by showing differences and similarities between milestones–schizotypy and milestones–schizophrenia associations. Its other strengths are a large homogeneous sample and prospectively collected data on several early motor developmental milestones. Included scales on schizotypy have been shown to be useful in the identification of intermediate psychosis phenotypes.^9,37^ In the current study, those with missing data differed from participants with complete data on gender and parental age. This is similar to a previous study on schizotypy scales in NFBC 1966, where they differed on gender and educational level. The additional analysis conducted in this previous study showed that nonparticipation was unlikely to influence the results.^37^ Therefore, as we utilize the same sample, attrition should not largely affect the results of the current study. Parental psychosis had some effect on milestones–scales associations, which varied depending on whether schizophrenia cases were included or not. Previously in NFBC 1966, Keskinen et al^32^ found that in both groups with and without parental psychosis delay in achievement of early motor developmental milestones was associated with increased risk of schizophrenia. Still, in the current article we took in consideration the effect of parental psychosis in the analyses by adding it as a covariate. As additional limitation, it should be mentioned that early motor developmental milestones are also associated with low cognitive functioning,^50^ alcohol dependence,^51^ and a level of neuroticism.^45^ Therefore, these milestones may lack specificity as risk factors for schizotypy or schizophrenia.^52^ Therefore, these limitations could have resulted in both under- and overestimation of the studied associations. While we included some of potential factors as covariates, the lists could have been broader including, eg, early neglect or reduced stimulation during infancy.^45^ However, these covariates were not available.

## Conclusions

The findings support an association of adult schizotypy traits with early motor developmental milestones and, thus, indirectly its link with schizophrenia. However, the results varied by the type of milestones, scales, and gender. Neurobiological explanations of milestone–trait association require further examination.

## Supplementary Material

Supplementary data are available at *Schizophrenia Bulletin* online.

Supplementary Flow Chart Of The Study

supplementary Table 1

Supplementary Table 2

Supplementary Table 3

Supplementary Table 4

## Funding

This work was supported by funding from the University of Oulu (65354 and 24500325); Oulu University Hospital (2/97, 8/97); the Ministry of Health and Social Affairs (23/251/97, 160/97, 190/97); the National Institute for Health and Welfare Helsinki (54121); the Regional Institute of Occupational Health, Oulu, Finland (50621, 54231); the People Programme (Marie Curie Actions) of the European Union’s Seventh Framework Programme FP7/2007–2013 under REA agreement (316795); the Wellcome Trust (201486/Z/16/Z); the Academy of Medical Sciences, UK (80354); and the Academy of Finland (303696 and 268336).
