# Supplementary figures and images for: Early Motor Developmental Milestones and Schizotypy in the Northern Finland Birth Cohort Study 1966
Source: Schizophr Bull. 2017 Dec 9;44(5):1151–8. doi: 10.1093/schbul/sbx165 (PMC6101480; doi:10.1093/schbul/sbx165)

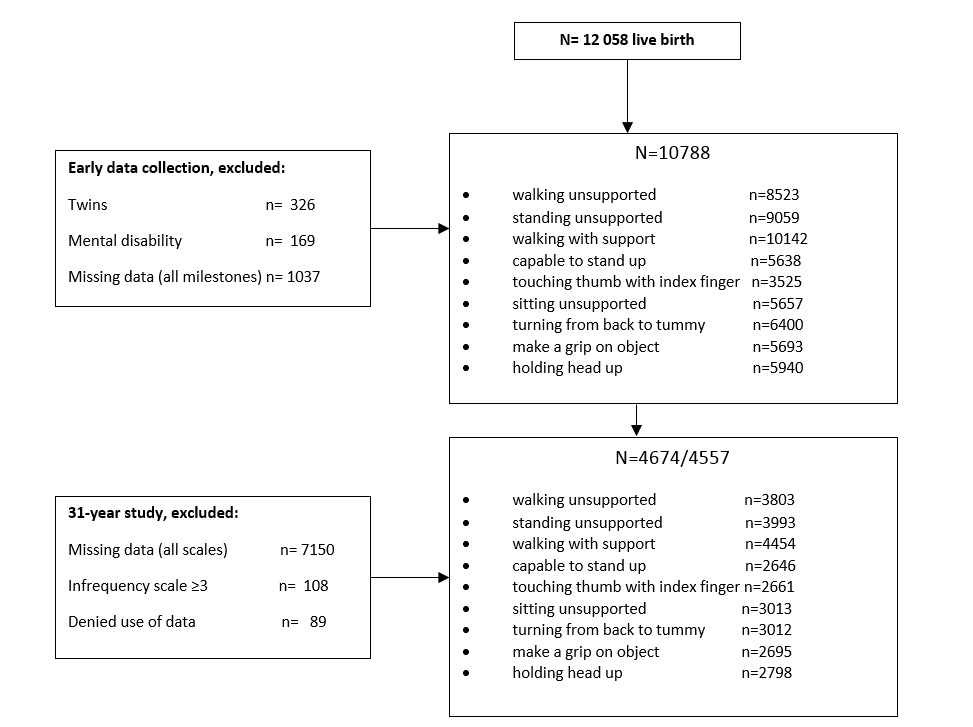

Supplement: Supplementary Flow Chart Of The Study [file sbx165_suppl_supplementary_flow_chart_of_the_study.jpeg]
