# Supplementary material for: Early Motor Developmental Milestones and Schizotypy in the Northern Finland Birth Cohort Study 1966
Source: Schizophr Bull. 2017 Dec 9;44(5):1151–8. doi: 10.1093/schbul/sbx165 (PMC6101480; doi:10.1093/schbul/sbx165)
Supplement: supplementary Table 1 [file sbx165_suppl_supplementary_table_1.doc]

**Supplementary Table 1 Sample characteristics by gender with schizophrenia cases included**

|  | **Women**  **(N=2602)^b^** |  | **Men**  **(N=2072)^b^** |  |  |
| --- | --- | --- | --- | --- | --- |
|  | **n** | **mean (SD)^a^ or %** | **n** | **mean (SD) or %** | **p-value** |
| ***Early-life characteristics*** | | | | | |
| **Parental psychosis**   - **Yes** - **No** | 167  2435 | 6.4%  93.6% | 105  1967 | 5.1%  94.9% | 0.051 |
| **Paternal age** | 2526 | 31.25 (7.23) | 2016 | 31.21 (7.24) | 0.846 |
| **Maternal age** | 2602 | 27.67 (6.68) | 2072 | 27.85 (6.71) | 0.359 |
| **Residence at birth**   - **Urban** - **Rural** | 805  1797 | 30.9%  69.1% | 605  1467 | 29.2%  70.8% | 0.200 |
| **Father’s social class**   - **High** - **Low** | 1180  1422 | 45.3%  54.7% | 1165  907 | 56.2%  43.8% | 0.286 |
| ***Early motor developmental milestones*** | | | | | |
| **Walking unsupported** | 2111 | 11.47 (1.49) | 1692 | 11.50 (1.52) | 0.477 |
| **Standing unsupported** | 2228 | 10.38 (1.33) | 1765 | 10.45 (1.31) | 0.089 |
| **Walking with support** | 2480 | 9.19 (1.42) | 1974 | 9.16 (1.41) | 0.504 |
| **Capable to stand up (lift themselves up)** | 1468 | 8.48 (1.37) | 1178 | 8.38 (1.35) | 0.043 |
| **Touching thumb with index finger (like a tweezer)** | 947 | 7.48 (1.32) | 740 | 7.46 (1.35) | 0.798 |
| **Sitting unsupported** | 1493 | 7.21 (1.11) | 1168 | 7.23 (1.14) | 0.581 |
| **Turning from back to tummy** | 1636 | 4.39 (1.08) | 1377 | 4.34 (1.06) | 0.273 |
| **Making a grip on object (grab object)** | 1476 | 3.23 (0.69) | 1219 | 3.24 (0.69) | 0.544 |
| **Holding the head up** | 1533 | 2.12 (0.72) | 1265 | 2.15 (0.73) | 0.261 |
| ***Schizotypal traits*** | | | | | |
| **Perceptual Aberration Scale (PAS)*** | 2586 | 2.60 (3.38) | 2060 | 1.99 (2.99) | p< 0.001 |
| **Physical Anhedonia Scale (PhAS)** | 2591 | 12.61 (5.82) | 2061 | 17.85 (7.24) | p< 0.001 |
| **Social Anhedonia Scale (SAS)*** | 2586 | 8.10 (4.76) | 2058 | 11.02 (5.94) | p< 0.001 |
| **Bipolar II Scale (BIP2)** | 2591 | 10.50 (3.74) | 2063 | 10.72 (3.86) | 0.050 |
| **Hypomanic Personality Scale (HPS)** | 2582 | 11.90 (7.01) | 2054 | 10.50 (6.92) | p<0.001 |
| **Schizoidia Scale (SCHD)** | 2590 | 2.80 (1.38) | 2064 | 2.25 (1.41) | p<0.001 |

*Not standardized scales; a: Standard Deviation; b: number of participants who had data on at least one scale and one milestone
