# Supplementary material for: Early Motor Developmental Milestones and Schizotypy in the Northern Finland Birth Cohort Study 1966
Source: Schizophr Bull. 2017 Dec 9;44(5):1151–8. doi: 10.1093/schbul/sbx165 (PMC6101480; doi:10.1093/schbul/sbx165)
Supplement: Supplementary Table 2 [file sbx165_suppl_supplementary_table_2.doc]

**Supplementary Table 2 Psychoses cases distribution (N=117)**

| Diagnosis | N (%) |
| --- | --- |
| Schizophrenia narrow   - paranoid - atypical - latent - simple - other specified schizophrenia - unspecified | 34 (29.1%)   - 15 - 8 - 1 - 1 - 1 - 8 |
| Schizophrenia spectrum   - schizoaffective disorder - delusional disorder | 15 (12.8%)   - 5 - 10 |
| Bipolar disorder with psychotic features | 12 (10.3%) |
| Major depressive episode with psychotic features | 27 (23.1%) |
| Brief psychosis | 8 (6.8%) |
| Other non-organic psychoses | 21 (17.9%) |
