# Supplementary material for: Early Motor Developmental Milestones and Schizotypy in the Northern Finland Birth Cohort Study 1966
Source: Schizophr Bull. 2017 Dec 9;44(5):1151–8. doi: 10.1093/schbul/sbx165 (PMC6101480; doi:10.1093/schbul/sbx165)
Supplement: Supplementary Table 3 [file sbx165_suppl_supplementary_table_3.doc]

**Supplementary Table 3**. Polychoric correlations between parental psychosis or place of residence or father’s SES and early motor developmental milestones or schizotypy scales

| **Correlation** | **Correlation coefficient** | **p-value** | **Correlation coefficient** | **p-value** |
| --- | --- | --- | --- | --- |
| *Parental psychosis* | Schizophrenia excluded | | Schizophrenia included | |
| Walking unsupported^a^ | 0.04 | 0.01 | - | - |
| Standing unsupported^a^ | 0.03 | 0.03 | - | - |
| PAS^a^ | -0.03 | 0.02 | 0.04 | 0.001 |
| SAS^a^ | - | - | 0.03 | 0.01 |
| PhAS^a^ | - | - | 0.04 | 0.01 |
| SCHD^a^ | - | - | 0.04 | 0.01 |
| BIP2^a^ | - | - | 0.04 | 0.01 |
| HPS^a^ | - | - | 0.03 | 0.01 |
| *Place of residence* | | |  |  |
| Walking unsupported^a^ | -0.02 | 0.05 | - | - |
| Walking with support^a^ | -0.04 | <0.001 | - | - |
| Capable to stand up (lift themselves)^a^ | -0.04 | 0.02 | - | - |
| Making a grip on object (grab object)^a^ |  |  | -0.04 | 0.01 |
| Touching thumb with index finger (like a tweezer)^a^ | 0.06 | 0.008 | - | - |
| Holding the head up ^a^ | 0.04 | 0.04 | - | - |
| SCHD^a^ | -0.04 | 0.05 | - | - |
| Walking with support ^b^ | -0.04 | 0.006 | -0.03 | 0.03 |
| Capable to stand up (lift themselves)^b^ | - | - | -0.04 | <0.001 |
| Sitting unsupported ^b^ | - | - | -0.07 | <0.001 |
| Making a grip on object (grab object)^b^ | - | - | -0.10 | <0.001 |
| Touching thumb with index finger (like a tweezer) ^b^ | 0.06 | 0.005 | -0.05 | <0.001 |
| Turning from back to tummy^b^ |  |  | -0.07 | <0.001 |
| Holding the head up ^b^ | 0.06 | 0.001 | -0.09 | <0.001 |
| SAS^b^ | -0.04 | 0.03 | - | - |
| SCHD^b^ | -0.04 | 0.05 | - | - |
| BIP2^b^ | -0.04 | 0.05 | - | - |
| *Father’s SES* | | |  |  |
| Walking unsupported^a^ | -0.05 | <0.001 | 0.03 | 0.02 |
| Standing unsupported^a^ | 0.04 | -0.03 | - | - |
| Walking with support^a^ | -0.07 | <0.001 | - | - |
| Capable to stand up (lift themselves)^a^ | -0.07 | <0.001 | - | - |
| Turning from back to tummy^a^ | -0.09 | <0.001 | - | - |
| Making a grip on object (grab object)^a^ | -0.06 | 0.004 | - | - |
| SAS^a^ | -0.09 | <0.001 | - | - |
| PhAS^a^ | -0.08 | 0.003 | - | - |
| SCHD^a^ | -0.05 | 0.01 | - | - |
| BIP2^a^ | -0.07 | 0.003 | - | - |
| Walking unsupported ^b^ | -0.03 | 0.05 | - | - |
| Standing unsupported^b^ | -0.04 | 0.006 | - | - |
| Walking with support ^b^ | -0.07 | <0.001 | - | - |
| Capable to stand up (lift themselves)^b^ | -0.07 | <0.001 | - | - |
| Sitting unsupported ^b^ | -0.05 | 0.04 | - | - |
| Turning from back to tummy ^b^ | -0.06 | <0.001 | - | - |
| Making a grip on object (grab object) ^b^ | -0.06 | 0.002 | - | - |
| PAS ^b^ | -0.04 | 0.02 | - | - |
| SAS ^b^ | -0.07 | 0.002 | - | - |
| BIP2 ^b^ | -0.07 | 0.001 | - | - |

a: in men; b: in women; - not reported as not significant
