# Supplementary material for: Early Motor Developmental Milestones and Schizotypy in the Northern Finland Birth Cohort Study 1966
Source: Schizophr Bull. 2017 Dec 9;44(5):1151–8. doi: 10.1093/schbul/sbx165 (PMC6101480; doi:10.1093/schbul/sbx165)
Supplement: Supplementary Table 4 [file sbx165_suppl_supplementary_table_4.doc]

**Supplementary Table 4 Multivariate linear regression analyses of early motor developmental milestones and schizotypy scale in the samples with excluded cases of parental psychoses. Adjusted for** parental age, place of residence and father’s SES

| Scale/Milestone | Schizophrenia cases included | | | Schizophrenia cases excluded | | |
| --- | --- | --- | --- | --- | --- | --- |
| **PAS*** | B | 95% CI | p value | B | 95% CI | p value |
| Walking with support^a^ | 0.11 | 0.01-0.21 | 0.025 | **0.09** | **-0.01-0.18** | **0.070** |
| Capable to stand up (lift themselves)^a^ | **0.13** | **-0.02-0.27** | **0.085** | 0.06 | -0.07-0.20 | 0.368 |
| Touching thumb with index finger (like a tweezer)^a^ | 0.26 | 0.09-0.43 | 0.002 | 0.23 | 0.07-0.39 | 0.004 |
| **PhAS** |  |  |  |  |  |  |
| Capable to stand up (lift themselves)^a^ | 0.41 | 0.09-0.74 | 0.013 | **0.36** | **0.03-0.69** | **0.033** |
| Sitting unsupported^a^ | 0.41 | 0.03-0.79 | 0.037 | 0.36 | -0.02-0.75 | 0.064 |
| Turning from back to tummy^b^ | 0.34 | 0.07-0.60 | 0.014 | 0.36 | 0.09-0.63 | 0.008 |
| **SAS*** |  |  |  |  |  |  |
| Sitting unsupported^b^ | 0.26 | 0.04-0.49 | 0.021 | 0.25 | 0.03-0.47 | 0.023 |
| Turning from back to tummy^b^ | 0.33 | 0.10-0.55 | 0.004 | 0.32 | 0.10-0.54 | 0.005 |
| **SCHD** |  |  |  |  |  |  |
| Touching thumb with index finger (like a tweezer)^a^ | 0.09 | 0.01-0.16 | 0.026 | 0.08 | 0.00-0.15 | 0.038 |
| Turning from back to tummy^a^ | 0.12 | 0.05-0.20 | 0.002 | **0.10** | **0.02-0.17** | **0.012** |
| **BIP2** |  |  |  |  |  |  |
| Walking unsupported^a^ | -0.13 | -0.26- - 0.00 | 0.047 | -0.15 | -0.28- -0.02 | 0.028 |
| Making a grip on object (grab object)^a^ | **-0.30** | **-0.60-0.05** | **0.093** | -0.25 | -0.58-0.08 | 0.137 |

*not transformed, adjusted for birth weight; a: in men, b: in women**; changes in significant associations between original scales analysis with and without individuals with history of parental psychosis are highlighted in bold**
